# OBESITY PREVALENCE AND METABOLIC SYNDROME IN A PARK USERS

**DOI:** 10.1590/S0102-6720201500S100010

**Published:** 2015-12

**Authors:** Maíra Danielle Gomes de SOUZA, Lucio VILAR, Cinthia Barbosa de ANDRADE, Raíssa de Oliveira e ALBUQUERQUE, Lúcia Helena de Oliveira CORDEIRO, Josemberg Marins CAMPOS, Álvaro Antônio Bandeira FERRAZ

**Affiliations:** 1 Postgraduate Program in Surgery; 2 Department of Surgery and Clinical Medicine of the Federal University of Pernambuco, Recife, PE, Brazil

**Keywords:** Obesity, Overweight, Diabetes mellitus, Metabolic syndrome

## Abstract

***Background* -:**

Overweight and obesity are associated with metabolic syndrome and abdominal obesity, thereby increasing the risk of type 2 diabetes mellitus and cardiovascular diseases. In Brazil, there are still no precise data on the prevalence of these disorders, especially among individuals who carry out some kind of physical activity in public spaces and there are no education and prevention programs for obesity.

***Aim*:**

To investigate the prevalence of metabolic syndrome and obesity among park users.

***Methods*:**

A prospective, cross-sectional, descriptive study was conducted with 619 individuals assessed and stratified by profile according to a specific protocol. The group was characterized as follows: female (50.1%) and mean age =50.6±14.8, with predominance of individuals aged between 50 and 59 years (26.8%) and with higher education (68%) and a household income of between 4 and 10 minimum wages (29.2%).

***Results*:**

Regular physical exercise was reported by 78% of the individuals and it was found that 70.7% were nevertheless of above normal weight: 45% overweight and 25.7% obese, of whom 20.7% had obesity grade I, 3.9% grade II and 1.1% grade III. The prevalence of metabolic syndrome was 4.3%, mostly in men (6.3%). Arterial hypertension and type 2 diabetes mellitus were detected in 17.8% and 5.5%, respectively. In view of the influence of obesity on the occurrence of type 2 diabetes mellitus and metabolic syndrome, it was found that this association was not significant for the two conditions (p=0.014 and 0.017, respectively).

**Conclusion:**

: The findings demonstrate a high prevalence of overweight and obesity in the studied population, and metabolic syndrome in 4.3%, despite the fact that 70% reported engaging in regular physical activity.

## Abstract (pt)

***Racional*:**

Sobrepeso

e obesidade estão associados à síndrome metabólica e obesidade abdominal, aumentando o risco de diabete melito tipo 2 e doenças cardiovasculares. No Brasil, ainda não há dados precisos sobre a prevalência destas alterações, principalmente entre pessoas que estão realizando algum tipo de atividade física em ambientes públicos; também não há programas de educação e prevenção da obesidade.

***Objetivo*:**

Investigar a prevalência da obesidade e síndrome metabólica em frequentadores de um parque em Recife.

***Métodos*:**

Realizou-se um estudo prospectivo, transversal e descritivo, onde 619 indivíduos foram avaliados e estratificados por perfil definido em protocolo específico, apresentando as seguintes características: sexo feminino (50,1%) e média de idade = 50,6 ± 14,8, havendo predomínio entre 50 e 59 anos (26,8% dos casos) e de indivíduos com ensino superior (68%) e renda familiar entre 4 e 10 salários mínimos (29,2%).

***Resultados*:**

Prática regular de exercícios físicos foi relatada por 78% das pessoas e foi constatado ainda que 70,7% apresentavam excesso de peso: 45% com sobrepeso e 25,7% obesidade, dos quais 20,7% com obesidade grau I, 3,9% com grau II e 1,1% com grau III. A prevalência de síndrome metabólica foi de 4,3%, com predomínio nos homens (6,3%). Hipertensão arterial e diabetes mellitus tipo 2 foram detectados em 17,8% e 5,5%, respectivamente. Devido a influência da obesidade na ocorrência de diabetes mellitus 2 e síndrome metabólica, foi constatado que esta associação não é significativa para as duas condições (p=0,014 e 0,017, respectivamente).

**Conclusão:**

: Os achados demonstram elevada prevalência de sobrepeso e obesidade da população avaliada, e síndrome metabólica em 4,3%, apesar do relato de que 70% dos transeuntes realizavam atividade física regular.

## INTRODUCTION

Overweight and obesity affect around two billion people around the world and are considered to be pandemic^17^^,^ 18. Brazil ranks fifth in the world, with around 60 million overweight and 22 million obese individuals, representing 17% of the population^16^. This leads to increased mortality as a result of the risk of developing other diseases, such as type 2 diabetes mellitus (DM2), systemic arterial hypertension (SAH), dyslipidemia, sleep apnea, cardiovascular diseases and certain kinds of cancer^9^^,^ 13. The increasing prevalence of DM2^16^^,^ 26 indicates the need for early detection and adequate control of these disorders.

Metabolic syndrome (MS) is a complex of interrelated risk factors for cardiovascular disease and DM2, including hyperglycemia, SAH, dyslipidemia and abdominal obesity^1^. It is estimated that MS affects 20% to 25% of the adult population and prevalence is on the increase, owing to obesity and a sedentary lifestyle^1^^,^ 11, affecting as many as 42% of individuals aged over 60 years^1^^,^ 11. Taken separately, the components of MS increase the risk of DM2, cardiovascular disease and mortality from all causes, although full-blown MS carries a risk higher than that of all the individual components combined^8^. It has been reported that the association between MS and cardiovascular disease increases total mortality by a factor of 1.5 and cardiovascular mortality by a factor of 2.5^21^. People with MS have five times greater risk of developing DM2^11^. In fact, MS is found in 42-64% of individuals with pre-diabetes and 78-84% of those with DM2^12^. In these individuals cardiovascular morbimortality is significantly higher when MS is present^12^. 

Despite the importance of MS in the context of metabolic and cardiovascular diseases, few studies in Brazil have assessed its prevalence and determining factors, limiting the quality of information available on the scale of the problem in the country. A recent systematic review of ten studies, none of them involving adults from the Northeast region, found prevalence of MS of around 29%^23^.

Weight loss is essential for obese individuals, whether or not they have MS. Physical exercise and dieting are thus very important for treatment. Health services should encourage healthy habits, and, in view of the low public investment training in health education, there is a need to develop strategies to improve performance and tract obesity and its comorbidities, in which a trained multidisciplinary team plays a fundamental role, thereby minimizing costs to the public health system. 

The main aim of the present study was to assess the prevalence of obesity and associated diseases, such as DM2, SAH and MS in a population of park users in the city of Recife, PE, Brazil.

## METHODS

The present study was approved by the Ethics Committee of the Federal University of Pernambuco's Center for Health Sciences (CEP/CCS/UFPE) (n° 915.390/2014) CAAE: 38937514.2.0000.5208. It adopted a prospective, cross-sectional, descriptive and quantitative approach and was a population-based epidemiological study conducted in the Jaqueira Park, a leisure area in Recife, frequented daily by around 3000 individuals. The sample comprised 619 individuals, who were assessed and stratified according to profile. Assuming a confidence level of 95%, a sample error of 5% and the number of visitors to the park per day, a sample size of 341 is considered representative.

Six interventions were carried out focusing primarily on providing guidance to the population with regard to risk factors for obesity, SAH, DM2 and MS. "Tests" were carried out (measurement of arterial pressure, weight and height; body mass index [BMI], abdominal perimeter and capillary glycemia) and a standardized questionnaire was applied covering age, sex, level of education, household income, frequency of physical exercise. The interviews were conducted by an appropriately trained multidisciplinary team. The park users who presented with some alteration in these parameters were advised to attend the family health unit closest to their place of residence or consult a specialist.

Individuals of both genders aged 18 years or over were included in the study. Exclusion criteria included pregnancy and some kinds of mental illness that might impede understanding of the procedures carried out. 

### Dependent variables

### Metabolic syndrome

The criteria followed were those established by the International Diabetes Federation, including abdominal obesity in South Americans (abdominal circumference ≥90 cm in men and ≥80 cm in women), combined with two or more parameters: SAH (systolic arterial pressure ≥30 mmHg or diastolic ≥85 mmHg); dyslipidemia and hyperglycemia (glycemia ≥100 mg/dl on fasting or glycemia ≥200 mg/dl after eating)^11^.

### Obesity

The body mass index (BMI) was used as a diagnostic parameter, in accordance with the criteria of the World Health Organization (WHO)^27^.

### Diabetes mellitus 

The diagnostic criterion followed the recommendations of the American Diabetes Association, namely, fasting glycemia ≥126 mg/dl or random glycemia ≥200 mg/dl, in combination with classic symptoms of the disease^2^. Hyperglycemia was defined as fasting glycemia ≥100 mg/dl or random glycemia ≥200 mg/dl^2^.

###  Systemic arterial hypertension (SAH) 

 Diagnosis was based on the recommendations contained in the 6^th^edition of the Brazilian Guidelines on Hypertension, in which SAH is classified into three stages according to systolic arterial pressure (SAP) and diastolic arterial pressure (DAP), respectively: stage 1 (140-159/90-99 mmHg); stage 2 (160-179/100-109 mmHg); and stage 3 (≥180/≥110 mmHg)^22^.

### Independent variables 

### Sociodemographic 

Gender, age, level of education (classified according to number of years of formal schooling) and household income (R$ - Brazilian reais). 

### Behavioral

Frequency of physical exercise.

### Statistical analysis 

The sample size was calculated using the Epi Info program Version 3.01, with a confidence level of 95%. A data bank was built up using Microsoft Excel and exported to SPSS for analysis. The profile of the participants was established by calculating percentage frequencies and drawing up respective frequency distributions for the factors under investigation (physical activity, distribution of BMI, MS, SAH and DM2). The chi-squared test was applied to compare the proportion and prevalence of comorbidities per study factor and also for independence, as a way of confirming the relation between obesity and DM2 and MS. In cases in which the association was significant, the odds ratios were calculated and used to measure the likelihood of obese individuals developing DM2 and MS compared to non-obese participants. All conclusions were obtained using a level of significance of 5% and confidence interval of 95% (p=0.05).

## RESULTS

### Characteristics of the participants

The profile distribution of the 619 participants showed that 50.1% were female and age varied from 18 to 88 years (mean 50.6±14.8), most falling in the 50-59 year age bracket (26.8% of cases). 

### Weight Status

It was found that 45% (n=276) of participants were overweight (BMI³25 and <30 kg/m^2^) and 25.7% (n=158) obese, of whom 20.7% (n=127) were classified as grade 1 (BMI between 30 and 34.9 kg/m^2^); 3.9% (n=24) as grade II (BMI between 35 and 39.9 kg/m^2^) and 1.1% (n=7) as grade III (IMC³40 kg/m^2^) (Table 1). The prevalence of obesity did not differ between men and women (27.1% vs. 24.4%; p=0.432) (Table 2).

**TABLE 1:** - Distribution of BMI, arterial pressure and glycemia

Study factor	n	%	p¹
BMI			
Underweight	7	1.1	<0.001
Normal weight	173	28.2	
Overweight	276	45.0	
Obese type I	127	20.7	
Obese type II	24	3.9	
Obese type III	7	1.1	
Arterial Pressure			
Excellent	172	28.2	<0.001
Normal	195	31.9	
Borderline	135	22.1	
Hypertension stage 1	85	13.9	
Hypertension stage 2	21	3.4	
Hypertension stage 3	3	0.5	
Glycemia			
³126	89	14.8	<0.001
<126	511	85.2	

¹p of chi-squared test for comparison of proportion (if p<0.05 the proportions are different)

**TABLE 2:** - Prevalence of comorbidities by socioeconomic factors

Study factor	Comorbidity			
	Obesity	Metabolic syndrome	Arterial hypertension	Diabetes mellitus
Gender				
Male	27.1	6.3	24.3	7.0
Female	24.4	2.3	11.4	2.7
p	0.432	0.017	<0.001	0.014
Age				
18-29 years	13.3	1.7	5.0	1.8
30-39 years	34.4	1.1	11.7	1.1
40-49 years	25.0	2.5	11.8	2.5
50-59 years	29.6	5.0	19.1	6.5
60-69 years	27.5	6.5	25.9	7.5
>=70 years	12.7	8.2	32.8	8.2
p	0.008	0.144	<0.001	0.093
Level of education				
No schooling	0.0	0.0	50.0	0.0
Primary comp/incomp	43.1	14.6	29.4	14.9
Secondary comp/incomp	29.6	4.3	20.1	5.9
Higher comp/incomp	22.6	2.9	15.4	3.2
p	0.005	<0.001	-	0.001
Household income				
1 MW	35.6	7.0	22.0	9.3
> 1-3 MW	32.0	6.5	26.6	7.3
4 -10 MW	17.6	2.3	14.9	3.0
10-20 MW	26.8	3.0	15.0	3.1
Over 20 MW	23.3	5.6	15.1	5.6
p	0.017	0.264	0.052	0.170

### Prevalence of MS and its components 

Twenty-six individuals with MS were identified (4.3% of the total), mostly male (6.3%). MS was also found to be more prevalent in the obese than the non-obese (9.8% vs. 2.4%; p<0.001, Table 3).

The prevalence of MS increased with age: 8.2% of patients ³70 years and 1.7% in the 18-29 year age bracket (Table 2). 

SAH, hyperglycemia and DM2 were also found to be present in 17.8%, 8.3% and 5.5% of cases, respectively (Tables 1 and3). Abdominal obesity, characterized by increased abdominal circumference, was the most prevalent component of MS, being found in 70.1% of men and 73.8% of women (Table 4). 

**TABLE 3:** - Distribution of metabolic syndrome by presence or absence of obesity and nutritional status vs glycemia

BMI	Metabolic syndrome	
	Yes	No
Not obese	11 (2.4%)	441 (97.6%)
Obese	15 (9.8%)	138 (90.2%)
Obese and not obese	26 (4.3%)	579 (95.7%)

Nutritional Status	Glycemia			
	<100	³100 e <126	³126 e <200	³200
Fasting	75 (76.5%)	15 (15.3%)	6 (6.1%)	2 (2.0%)
After eating	258(53.1%)	150 (30.9%)	57(11.7%)	21 (4.3%)
Random	3 (33.3%)	4 (44.4%)	4 (44.4%)	0 (0%)

p test of independence <0.001; Odds ratio=4.08 (1.89-8.58)

### Influence of demographic and anthropometric factors

While MS was found to be more prevalent in men (6.3% vs 2.3%; p=0.017), the occurrence of obesity did not differ between men and women (27.1% vs 24.4%; p=0.432) (Table 2). The components of MS found to be more frequent in men were DM2 (7.0% vs 2.7%; p=0.014) and SAH (24.3% vs 11.4%; p<0.001), while the frequency of abdominal obesity was similar for men and women (70.1 vs 73.8%; p=0.309) (Table 4).

**TABLE 4:** - Prevalence of increased abdominal circumference by gender and age bracket

Study factor	Increased abdominal circumference
Gender	
Male (%)	70.1
Female (%)	73.8
p^1^	0.309
Age bracket	
18-29 years (%)	31.7
30-39 years (%)	65.3
40-49 years (%)	71.4
50-59 years (%)	75.0
60-69 years (%)	88.0
³70 years (%)	84.1
p^1^	<0.001

¹Chi-squared test

So far as age bracket is concerned, obesity was more prevalent among those aged 30-39 years (34.4%), while the prevalence of MS increased with age, being low among those aged 18-29 years (1,7%) and higher in individuals aged over 70 (8.2%) (p=0.144). The prevalence of the components of MS (SAH, DM2 and increased abdominal circumference) also increased with age (Tables 2 and 4). 

### Influence of socioeconomic factors

The participants who had only primary school education were those who had the highest prevalence of the comorbidities under study (43.1% for obesity and 14.6% for MS). As for the influence of household income, the participant with up to one minimum wage were those who had the highest prevalence of obesity (35.6%) and MS (7%, Table 2).

### Influence of physical exercise 

With regard to physical exercise, the sedentary participants had a higher prevalence of obesity and metabolic syndrome (though not statistically significant) (Table 5).

**TABLE 5:** - Prevalence of obesity and metabolic syndrome by physical activity

Variable	Obesity	Metabolic syndrome
Physical exercise		
Yes	23.9	3.8
No	32.6	6.0
p¹	0.042	0.271
Frequency of physical activity (weekly)		
None	32.6	6.0
1-2 times	32.5	3.8
3 times	20.2	2.5
4-5 times	22.7	4.6
6-7 times	22.9	4.3
p-value¹	0.078	0.751

¹Chi-squared test

### Influence of obesity and abdominal circumference 

Glycemia levels 126 mg/dl were found to be significantly more prevalent in the obese individuals than in those that were not obese (24.3% vs 11.7%; p<0.001), with an odds ratio of 2.44 (IC=1.48-4.00) (Table 6), and in those with increased abdominal circumference (p<0.001, Table 6). The relation between metabolic syndrome and obesity was also found to be significant (p<0.001), with an odds ratio of 4.08 (CI=1.89-8.58, Table 3). 

**TABLE 6:** - Distribution of glycemia according to the presence or absence of obesity and abdominal circumference

BMI	Glycemia*	
	³ 126 mg/dL	< 126 mg/dl
Not obese	52 (11.7%)	394 (88.3%)
Obese	37 (24.3%)	115 (75.7%)
All	89 (14.9%)	509 (85.1%)

AC	Glycemia	
	³ 126 mg/dL	< 126 mg/dL
Normal	14 (6.9%)	190 (93.1%)
Increased	73 (18.8%)	315 (81.2%)
All	87 (14.7%)	505 (85.3%)

*Patients fasting and after eating; p for test of independence <0.001; Odds ratio=2.44 (CI=1.48-4.00)

AC= abdominal circumference; p for test of independence <0.001; Odds ratio=3.15 CI=1.67 - 6.01)

## DISCUSSION

The present study forms part of a broader project that aims to trace the epidemiological profile and prevalence of obesity and comorbidities among users of a park - chosen on account of the large numbers who visit it - both to engage in physical activities and walk. 

Of the participants, 72% presented with abdominal obesity, 45% overweight, 25.7% obesity, 17.8% SAH, 8.3% hyperglycemia, 5.5% DM2 and 4.3% MS. All these conditions were found to be significantly more prevalent in men, with the exception of obesity and abdominal obesity, whose prevalence was similar in both genders. MS and its components were significantly more frequent in obese individuals than in the non-obese and its prevalence increased notably with age.

Obesity is a major public health problem, with growing numbers of people affected and serious comorbidities and mortality associated with the condition^9^^,^ 13 ^,^ 17. A recent study examining the prevalence of obesity and overweight around the world found that, in Brazilian men and women aged over 20 years, the rates for overweight and obesity were 52.5% and 58.4%, respectively, while the corresponding prevalence for obesity alone was 11.7% and 20.6%^17^. Similar results have been reported by Ministry of Health studies, in which around half (50.8%) of the Brazilian population was found to be overweight **,** the proportion of individuals with BMI 25 kg/m^2^ increased from 42.7% in 2006 to 50.8% in 2013 (54.7% in men and 47.4% in women)^16^. There was also found to be a prevalence of obesity (BMI 30 kg/m^2^) of 17.5% (16.4% in men and 19.2% in women)^16^. This survey was conducted by VIGITEL (Telephone Surveillance of Risk and Protective Factors for Chronic Diseases) and the data were collected in 26 Brazilian state capitals and the Federal District^16^. Compared with the current state of affairs, these results call attention to the higher prevalence of obesity (25.7%) in both men (24.4%) and women (27%). This difference may be due to many people visiting the park to lose weight by engaging in physical activity. 

Data on the prevalence of MS are still limited in the country. A recent systematic review, involving nine cross-sectional studies, showed that its prevalence in Brazil varied from 14.9% to 65.3%, with the highest percentage found in the indigenous population of Rio Grande do Sul^23^. The mean prevalence was 29.8% in urban areas, 20.1% in rural areas and 41.5% among indigenous peoples, with an overall mean prevalence of 28.9% and 29.5%, according to criteria used to define MS^23^. 

The lower prevalence of MS found in the present study, compared with the Brazilian systematic review and that reported in some other countries^3^^,^ 4 ^,^ 5 ^,^ 7 ^,^ 15 ^,^ 19 ^,^ 24 ^,2526^ may be explained by the characteristics of the sample (1.7% aged<30 years) or, more likely, by the fact that levels of triglycerides and HDL-cholesterol were not included in the diagnosis of MS. Moreover, the fact that only 20% of the participants were fasting hindered the evaluation of glycemic status and certainly led to underestimation of the prevalence of hyperglycemia. It has been widely demonstrated that the occurrence of MS increases with age and is much less frequent in those aged under 30 years^5^^,^ 11 ^,^ 23 and this is in keeping with the findings of the present study. Only 17% of university students in Fortaleza, CE, Brazil were found to have MS, but the syndrome affected 33% of overweight individuals and 41.7% of those who were obese^6^. Of 321 overweight adolescents (10-16 years) in Botucatu, SP, Brazil, around 18% were found to have MS^20^. In the present study, only 1.7% of participants aged under 30 years presented with MS. 

DM2 is a serious condition associated with increased morbimortality and an estimated 4.9 million deaths in 2014^2^^,^ 10 ^,^ 21 ^,^ 26. In the same year, the IDF estimated that DM2 affected 387 million individuals around the world (a prevalence of 8.3%), with a projected 205 million new cases by 2035^10^. According to data from the latest VIGITEL study, the mean prevalence of diabetes reported in adults was 6.9% (6.5% in men and 7.2% in women) but increased with age, reaching 22.1% in the group 65 years^16^. Around 8% of those interviewed in Recife reported having received a medical diagnosis of DM2^16^. Overall, the prevalence of DM2 was higher (14.9%) in individuals with up to eight years of schooling. Of those interviewed, only 5.5% had DM2, but the disease affected 8.2% of individuals aged over 70 years. The fact that most of the participants had eaten, as noted above, certainly led to an underestimation of the occurrence of DM2. 

A high prevalence of diabetes was also found by a study conducted in 2008/2009 in Triunfo, a small city in the Sertão Region of Pernambuco. In fact, 27 (13.6%) of 198 were diagnosed as having DM2 (8.8% of men and 16.2 of women)^14^. In that study, 80% of participants had only primary school education and 81.3% a household income of less than one minimum wage^14^. In the present study, DM2 was also more prevalent among individuals with only primary school education (14.9%), but there was no significant difference with regard to monthly income. 

MS was also found to be more prevalent in the group aged over 70 years, increasing the risk of cardiovascular diseases for this age group, while obesity was more prevalent in those aged between 30 and 39 years. These findings show that increasingly younger people are being affected and at greater risk of developing MS. Participants with lower levels of education were those with the highest prevalence of comorbidities, especially SAH. Obesity was found to be more prevalent in participants with an income of up to one minimum wage, showing that the least privileged social strata are more prone to the disease, which may be due to greater consumption of cheaper, less healthy, high-calorie food products. 

Some limitations of the present study should be pointed out. The study setting did not allow for more complex tests (such as lipid levels) to be carried out and most participants were not fasting. Neither was it possible to conduct follow-up observations. 

Interventions such as the one carried out here should be encouraged and adopted by public institutions in other leisure areas in the country, resulting in greater education of a population that is often unaware of its true state of health and aiming primarily to prevent such problems.

## CONCLUSION

The findings show a high prevalence of overweight and obesity in the studied population and metabolic syndrome in 4.3%, despite 70% of participants reporting regular physical activity.
